# X-ray induced photodynamic therapy (PDT) with a mitochondria-targeted liposome delivery system

**DOI:** 10.1186/s12951-020-00644-z

**Published:** 2020-06-10

**Authors:** Xuefan Gu, Chao Shen, Hua Li, Ewa M. Goldys, Wei Deng

**Affiliations:** 1 College of Chemistry and Chemical Engineering, Xi’an Shiyou University, Xi’an, 710065 China; 2 ARC Centre of Excellence for Nanoscale Biophotonics, Graduate School of Biomedical Engineering, University of New South Wales, Kensington, NSW 2052 Australia; 3 Faculty of Science and Engineering, Macquarie University, Sydney, NSW 2109 Australia

**Keywords:** Photodynamic therapy, Liposome, Mitochondria-targeted, X-ray, Gold nanoparticles

## Abstract

In this study, we constructed multifunctional liposomes with preferentially mitochondria-targeted feature and gold nanoparticles-assisted synergistic photodynamic therapy. We systemically investigated the in vitro X-ray triggered PDT effect of these liposomes on HCT 116 cells including the levels of singlet oxygen, mitochondrial membrane potential, cell apoptosis/necrosis and the expression of apoptosis-related proteins. The results corroborated that synchronous action of PDT and X-ray radiation enhance the generation of cytotoxic reactive oxygen species produced from the engineered liposomes, causing mitochondrial dysfunction and increasing the levels of apoptosis.

## Background

Photodynamic therapy (PDT) is a noninvasive therapy which has been widely used for cancer treatment [1, 2]. In this approach, a PDT drug (referred to as photosensitiser) is administered and subsequently activated by illumination with visible light at the target site. The activated photosensitiser reacts with available oxygen to form reactive oxygen species (ROS), which induce tumour cell death and vascular shutdown. The therapeutic effect of PDT depends on generation of sufficient amounts of ROS. Singlet oxygen (^1^O_2_), as one of the primary cytotoxic ROS, is generated by energy transfer between the triplet excited state of photosensitizers and the ground state of O_2_ [3]. Since the generated ^1^O_2_ has a short lifetime (~ 40 ns) and diffusion radius (~ 20 nm), it would be desirable for a PDT drug to be located in an organelle that sensitively responds to toxic effects of ^1^O_2_ [4–7]. Among the organelles of mammalian cells, mitochondria (~ 900 nm wide) were intensively studied as a target site of ^1^O_2_ to enhance PDT effect since they are highly susceptible to oxidative damage induced by ^1^O_2_ [8–12]. Targeted delivery of ^1^O_2_ induces dysfunction of mitochondria, leading to apoptotic death of cancer cells [13, 14].

Because of the negative membrane potential of the mitochondrial inner membrane, the mitochondria targeting approaches used earlier were based on the conjugation of lipophilic cations to the drugs or drug delivery vehicles [15]. Representative cations that are capable of mitochondria targeting include guanidinium, bisguanidinium and triphenylphosphine (TPP) [16–18]. Due to the advantages of TPP and its derivatives over other targeting moieties (stability in biological systems, simultaneous lipophilic and hydrophilic properties, and the absence of light absorption or fluorescence in the visible or near-infrared spectral region) [19, 20], the TPP moiety has been frequently used for directing chemotherapeutics, imaging probes and photosensitizers to the mitochondria^[^ [21–23].

The utility of PDT in the clinic have been hampered by the difficulty in delivering sufficient light to deep tumours. For example, red light (620–750 nm) required to activate verteporfin (VP, a PDT drug) penetrates only up to 3 mm allowing to treat superficial lesions only. The X-ray-induced PDT has emerged as an attractive alternative as it is able to overcome limited penetration depth of activating light in traditional PDT [24–28]. Unlike visible or near-infrared (NIR) light, X-rays have excellent tissue penetration capability. This emerging approach can potentially expand the scope of PDT in clinical applications. The X-ray induced PDT also offers an innovative feature of precise synchronisation of radiation and PDT, which are both cytotoxic but via different mechanisms, offering opportunities for synergistic effects and enhanced therapeutic efficacy. As cytotoxic species can only be generated within the radiotherapy field, any toxicity to other healthy tissues is largely reduced [29].

Earlier studies in the area of X-PDT employed scintillating nanoparticles to convert X-ray photons to visible photons, activating a nearby photosensitiser to produce ROS [26, 30, 31]. By contrast, our group established that verteporfin can be directly activated by low dose X-ray radiation, which we utilised in X-ray triggered drug release and X-ray induced PDT [24, 32]. The X-ray-induced ROS generation from verteporfin is caused by energetic secondary electrons produced in the tissue during X-ray radiation and a contribution from photoexcitation by Cherenkov radiation [32]. Compared with scintillator-mediated X-ray PDT, our approach to X-PDT relies on clinically approved agents such as verteporfin and this will aid and accelerate clinical translation. Due to the hydrophobicity of verteporfin, it requires formulation within liposomes to be clinically applicable [33]. Our liposome formulation is specifically designed for verteporfin activation from X-ray triggering [24]. Table 1 compares different attributes of our liposome formulation, with published approaches to comparable triggerable liposomes [34–36] providing evidence of novelty of this work.

**Table 1:** Features of our liposomes v.s. other triggered liposome formulations

Attribute	This work	Ref. [34]	Ref. [35]	Ref. [36]
Liposome has one photosensitizer inside	+	+	+	+
Gold nanomaterials in the bilayer or the middle core	+	–	–	–
Liposome is destabilised (triggered) by ROS generated in the liposome	+	+	+	+
Liposome is destabilised (triggered) by ROS generated from radiation X-ray radiation	+	–	–	–
Free of chemodrug toxicity	+	–	–	–

**Scheme 1:**
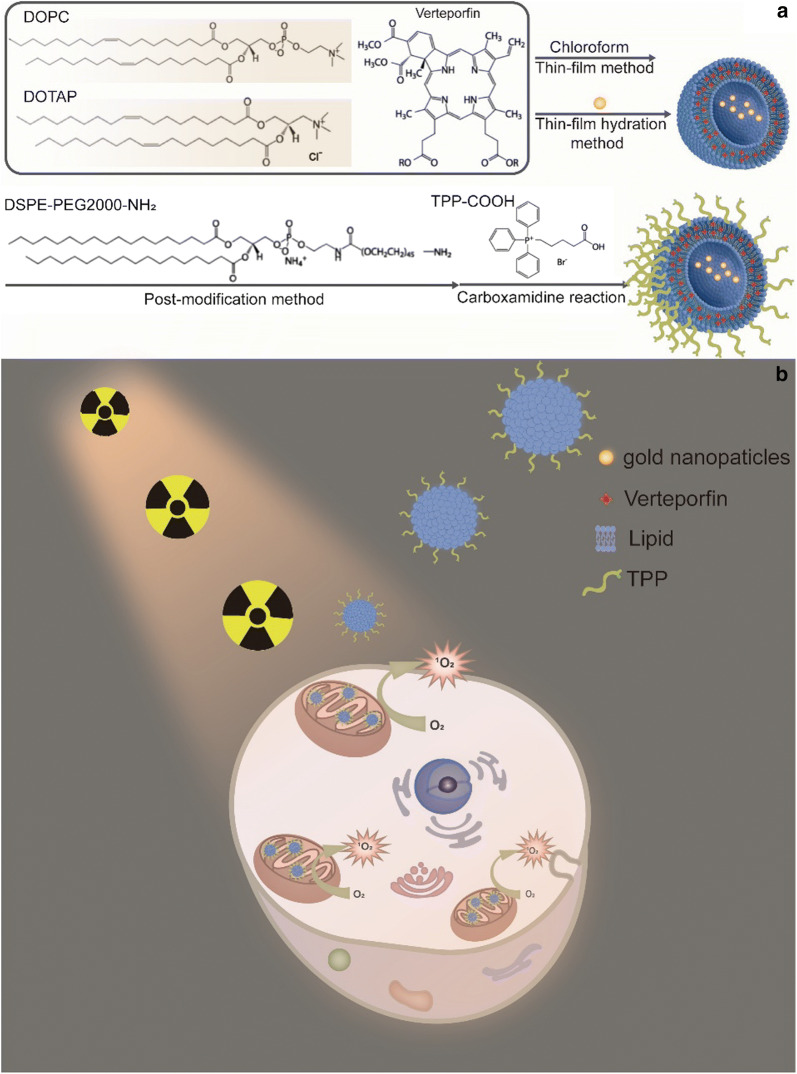
The schematic illustration of **a** the liposome formulation and **b** mitochondria-targeted X-ray induced PDT via the liposome delivery system

In this report we describe rationally designed mitochondria-targeted liposomes that carry and direct verteporfin to the mitochondria where a low dose X-ray radiation is able to trigger generation of cytotoxic ^1^O_2_ from verteporfin. Co-encapsulation of gold nanoparticles (10 nm) in the central core of the liposomes enhances ^1^O_2_ generation. Scheme 1 illustrates the formulation of the liposome system and the mechanism of mitochondria-targeted PDT. Subcellular localization of the liposomes, intracellular ^1^O_2_ generation, changes in mitochondrial membrane potential (Δ*ψ*_m_), cellular apoptosis/necrosis pathway and in vitro X-ray triggered PDT effect in human colorectal cancer cells (HCT116) were investigated.

## Materials and methods

### Materials

1,2-dioleoyl-3-trimethylammonium-propane(chloride salt) (DOTAP) (Catalogue No. 890890), 1,2-dioleoyl-sn-glycero-3-phosphocholine (DOPC) (Catalogue No. 850375), 1,2-distearoyl-sn-glycero-3-phosphoethanolamine-N-[amino(polyethylene glycol)-2000] (ammonium salt) (DSPE-PEG(2000)-NH_2_) (Catalogue No. 880128), chloroform (Catalogue No. C2432), verteporfin (Catalogue No. SML0534), dimethyl sulfoxide (DMSO) (Catalogue No. 472301), 10 nm Gold nanoparticles (Catalogue No. 752584), 5 nm Gold nanoparticles (Catalogue No. 752568), (4-Carboxybutyl)triphenylphosphonium bromide (TPP-COOH) (Catalogue No. 157945), N-Hydroxysuccinimide (NHS) (Catalogue No. 130672), N-(3-Dimethylaminopropyl)-N′-ethylcarbodiimide hydrochloride (EDC) (Catalogue No. E7750), McCoy’s 5A Medium (Catalogue No. M8403), 2-(N-Morpholino) ethanesulfonic acid (MES) (Catalogue No. M3671), Methanol (Catalogue No. 34860) were purchased from Sigma-Aldrich Pty Ltd. Singlet Oxygen Sensor Green (SOSG) (Catalogue No. S36002), Fetal Bovine Serum (FBS) (Catalogue No. 16000036), live cell imaging solution (Catalogue No. A14291DJ), NucblueTM live cell stain readyprobesTM reagent (Catalogue No. R37605), MitoTracker™ deep red FM (Catalogue No. M 22426), LysoTracker™ Red DND-99 (Catalogue No. L7528), Dulbecco’s Phosphate-Buffered Saline (DPBS) (Catalogue No. 14190144), RIPA Lysis and Extraction Buffer (Catalogue No. 89900), Halt™ Protease Inhibitor Cocktail (100X) (Catalogue No. 78438), PVDF Pre-cut Blotting Membranes (Catalogue No. LC2002), Blocker™ BSA (10X) in TBS (Catalogue No. 37520), NuPAGE™ 10% Bis–Tris Protein Gels (Catalogue No. NP0315BOX), Pierce™ 20X TBS Tween™ 20 Buffer (TBST, Cat No. 28360) and Pierce™ ECL Western Blotting Substrate (Cat No. 32109) were purchased from Thermo Fisher Scientific Inc. Cleaved Caspase-3 (Asp175) Rabbit mAb (Biotinylated) (Cat No. 9654S, Bax Antibody (Cat No. 2774S), Bad (D24A9) Rabbit mAb (Cat No. 9239S), Streptavidin-HRP (3999S) and Anti-rabbit IgG, HRP-linked Antibody (7074S) were purchased from Cell Signalling Technology. Apoptosis/necrosis/healthy detection kit (Catalogue No. PK-CA707-30018) and CellTiter 96^®^ AQueous one solution cell proliferation assay (Catalogue No. G3582) were purchased from Promega Corporation.JC-1-mitochondrial membrane potential assay kit (Catalogue No. ab113850) was purchased from Abcam Corporation. Eagle’s Minimum Essential Medium (Catalogue No. ATCC^®^30-2003) was purchased from ATCC.

## Methods

### Preparation of pure liposomes and liposomes incorporating gold nanoparticles and VP

Liposome-based nanocarriers in this work were prepared using a thin-film hydration method [24]. Briefly, 25 µL of DOTAP (100 mM) was mixed with 25 µL of DOPC (100 mM) dissolved in chloroform, followed by addition of 4 µL VP solution (3 mM, dissolved in DMSO). For the synthesis of pure liposomes and liposomes loaded with gold nanoparticles alone, VP were omitted in the mixture solution. The mixture solvent was then evaporated in a rotary evaporator (Rotavapor^®^ R-300, BÜCHI) for 10 min at 70 °C. The thin lipid film was formed around the wall of the flask and subsequently hydrated with PBS buffer (pH 7.4) (in the case of pure liposomes and liposomes incorporating VP only) or gold nanoparticle suspension (in the case of liposomes incorporating VP and gold nanoparticles) with vigorous stirring for 30 min until the suspension was homogenized (Vortexer, Heathrow Scientific). The hydrated lipid suspension was kept at room temperature for 3 h to allow the maximal swelling of liposomes. The suspension was then extruded eleven times through a 200 nm polycarbonate membrane in an automated extrusion equipment (NanoSizerTM AUTO, T&T Scientific Corporation, USA). The resultant suspension was stored at 4 °C.

### Liposome surface modification with TPP-COOH

PEGylated and TPP-conjugated liposomes were prepared by post-insertion of DSPE-PEG(2000)-NH_2_ micelles into preformed liposomes and subsequent EDC-NHS coupling method with slight modifications [37]. In brief, 500 μL of aforementioned liposomes and 50 μL of DSPE-PEG(2000)-NH_2_ (0.01 M in distilled water) co-incubated at 60 °C for 1 h, followed by centrifugation 3 times (10,000 rpm, 10 min) with Amicon Ultra 0.5 mL centrifugal filters (100 kDa) to remove the free molecules. After that TPP-COOH (10 mg) was dissolved in 1 mL MES buffer (pH = 6), followed by addition of a mixture of EDC and NHS (molar ratio, 1:1). The solution was kept at room temperature for 30 min under stirring. After raising pH to 7.0–7.4, 200 µL of prepared PEGylated liposome suspension was added into the activated TPP solution. The mixed solution was gently vibrated via orbital shaker for 4 h at room temperature. After reaction, the solution was washed with PBS buffer three times (11,000 rpm, 10 min each time) with Amicon Ultra centrifugal filters (100 kDa). The final liposome conjugate suspension was kept at 4 °C for future use.

### Characterisation of liposomes

The extinction spectra of pure gold colloidal solution and prepared liposome samples were measured using a spectrophotometer (Cary 5000 UV–Vis-NIR, Agilent Technologies). Size distribution and zeta potentials of liposomes were measured under a Zetasizer Nano-ZS from Malvern Panalytical Co. The morphology of liposomes was determined using Transmission Electron Microscopy (TEM). For TEM imaging, the liposome samples were prepared by placing a drop of suspension onto a copper grid and air dried, following negative staining for contrast enhancement. The air-dried samples were then imaged by a PHILIPS CM 10 system at an accelerating voltage of 100 kV. Images were captured with an Olympus Megaview G10 camera and iTEM software.

### Assessment of ^1^O_2_ generation from the liposome samples under X-ray radiation

^1^O_2_ generation was detected by using SOSG probe based on our previous work 1.8 µL of SOSG (500 μM) was mixed with 200 µL of liposome suspension, followed by X-ray radiation at different dosage (1 Gy, 2 Gy and 4 Gy). The irradiation of samples was carried out via a 320 kV cabinet X-Ray Irradiator (X-RAD 320, Precision X-Ray, Inc.). The fluorescence signal of SOSG (excitation/emission wavelength: 488/525 nm) was recorded before and after X-ray radiation in a spectrofluorometer (FluoroMax-4 HORIBA Scientific Co.)

### Cell culture and X-ray radiation on cells

Human colon adenocarcinoma (HCT116 cells) and normal human colon epithelial cells (CCD841 CoN) were purchased from the American Type Culture Collection. HCT116 cells were cultured in McCoy’s 5 A (modified) medium and CCD841 CoN cells were cultured in EMEM. All culture media were supplemented with 10% FBS and 1% antibiotic–antimycotic. The flasks were incubated at 37 °C in the presence of 5% CO_2_ humidified air. The cells were seeded at appropriate dilutions into glass-bottom petri dishes for cell imaging or 96-well plates for cell viability assays. For X-ray radiation experiments, the cells were radiated by using the same X-ray irradiator as described in the ^1^O_2_ generation assessment.

### Imaging and analysis of cellular uptake of the liposomes

HCT116 cells (3 × 10^4^ cells/mL) were seeded in glass-bottom petri dishes and incubated at 37 °C for 48 h before treatment with the liposome suspension. After removing the culture medium, the cells were incubated with the liposome suspension (250 µM) in culture media for 1 h, 2 h and 4 h. The cells were then washed with PBS buffer (pH 7.4) three times to remove free liposomes. To assess the cellular uptake activity of the liposomes, the cells were stained with NucBlueTM Live ReadyProbesTM reagent for 20 min before imaging. The cells were imaged using an Olympus FV3000 confocal laser scanning microscopy system. A laser source at 405 nm was used for the excitation of VP.

For flow cytometry measurements, after incubation with the liposome suspension, the cells were washed three times with Dulbecco’s phosphate buffered saline, trypsinized, centrifuged at 3000 rpm for 3 min, and resuspended in DPBS. The cells were then analyzed using a flow cytometer (LSRFortessa™ X-20 cell analyser, BD Biosciences) equipped with a violet laser. FlowJo software was used for further analysis of FCM data.

### Intracellular ^1^O_2_ detection

HCT116 cells were cultured in glass-bottom petri dishes at a concentration of 1 × 10^5^ cells/mL. The cells were then treated with different liposome samples (250 µM) in culture medium for 3 h at 37 °C, followed by addition of SOSG solution (50 µM) for further 1 h incubation). After washed with fresh medium, the cells were irradiated with X-rays at different doses. SOSG fluorescence signal was imaged under FV3000 confocal laser scanning microscope. A laser at 488 nm was used for SOSG excitation. Quantitative analysis of SOSG signal was conducted by using ImageJ software, which indicated the intracellular ^1^O_2_ level generated under different experimental conditions.

### Mitochondrial localization of TPP-conjugated liposomes

HCT116 cells were seeded on a glass-bottom petri dish at a concentration of 1 × 10^5^ cells/mL for 48 h. After that the cells were incubated with liposome suspension for 4 h. After being washed two times with PBS, the cells were co-stained with Mitotracker Red (0.2 μM) and NucBlueTM Live ReadyProbesTM reagent in live cell imaging solution at 37 °C for 30 min. The cells were then washed twice with PBS and kept in live cell imaging solution for confocal imaging under FV3000 confocal laser scanning microscope. Laser sources at 405 nm and 561 nm were used for the capture of nucleus, VP and MitoTracker, respectively. Colocalization between TPP-conjugated liposomes and lysosomes was also verified by using LysoTracker™ Red DND-99 (0.2 μM) under the same experimental conditions, instead of MitoTracker™ Red.

### Apoptosis and necrosis assay

The apoptosis and necrosis assays were carried out using an apoptosis/necrosis/healthy detection kit. The kit allows simultaneous imaging of apoptotic (green), necrotic (red) and healthy cells (blue). HCT116 cells (1 × 10^5^ cells/mL) were first seeded onto glass-bottom petri dishes in the culture medium and then incubated with different liposome suspension for 4 h. After being washed with fresh medium, the cells were irradiated with 4 Gy X-ray and kept incubation for another 48 h. The cells were washed twice with PBS and co- stained with Hoechst33342, FITC-Annexin V, and Ethidium Homodimer III (EthD-III) provided by the kit in binding solution (5X dilution) at room temperature for 15 min in darkness. Multiple fluorescence images were taken using three filters: (1) Hoechst33342 (*λ*_abs_/*λ*_em_ = 350/461), indicating healthy cells; (2) FITC (*λ*_abs_/*λ*_em_ = 492/514 nm), representing apoptosis, and (3) ethidium homodimer *λ*_abs_/*λ*_em_ = 528/617 nm), indicating necrosis.

### Cell viability assays after X-ray induced PDT

Cells (1 × 10^4^/mL) were seeded onto 96-well plates and cultured for 48 h at 37 °C. When the cells reached 70% confluency, they were divided into four groups: control cells without treatment, cells incubated with the liposomes alone, cells treated with X-ray alone and cells treated with X-ray induced PDT. At 24 h post treatment, cellular cytotoxicity was assessed using the Cell Viability Assay Kit, MTS (Promega Corporation, USA) according to its protocol. The MTS assays were carried out under a plate reader (SpectraMax MiniMax 300 Imaging Cytometer, Molecular Devices). Cell viability was calculated as a percentage of the absorbance of the untreated control cells that was set to 100%.

### Assessment on mitochondrial membrane potential (Δ*ψ*_m_)

After HCT116 cell seeding, they were respectively incubated with liposome-TPP conjugates and non-conjugated liposomes in the cell medium for 4 h, followed by X-ray radiation at 4 Gy. At 24 h post treatment, cells were stained with 5 µM JC-1 probe at 37 °C for 15 min before fluorescence imaging. JC-1 fluorescence signal was captured at 590 ± 17 nm (red aggregates) and 530 ± 15 nm (green monomers), respectively. The Δ*ψ*_m_ analysis was conducted using ImageJ software to calculate the ratio of red-to-green channel intensities.

### Western blotting analysis

HCT116 cells were treated with TPP-conjugated liposomes for 4 h, X-rays only or with the combination of liposomes and X-ray treatment. After treatments the cells were washed twice with PBS and lysed with the RIPA buffer supplemented with a protease inhibitor cocktail (addition of protease inhibitor to the RIPA buffer immediately before use) according to the manufacturer protocol (ThermoFisher Scientific Australia). In Brief, 250 µL of the mixture was added to the cell pellet, followed by shaking the cell pellet gently for 15 min on ice. The cell mixture was centrifuged at ~ 14,000×*g* for 15 min to pellet the cell debris. The supernatant (extracted proteins) was transferred to a new tube for gel running. The extracted proteins were loaded in the wells of the Bis–Tris protein gel. After separation the proteins were transferred to PVDF membranes. The membranes were blocked with a blocking buffer (Thermo Fisher Scientific) for 1 h at room temperature and incubated with primary antibodies against Bax, BAD and cleaved caspase-3 at 4 °C overnight. All primary antibodies were diluted 1:1000. After washing with TBST 3 times, the membranes were incubated with the corresponding HRP-conjugated secondary antibodies for 1 h at room temperature. All secondary antibodies were diluted 1:1000. After washing with TBST 3 times, the membranes were visualises using Western Blotting Substrate on a ChemiDoc™ MP Imaging System (Bio-Rad Laboratories, Inc., USA). Quantifications of western blots was conducted by using ImageJ software.

### Statistical analysis

All quantitative data are shown as mean ± SD, n ≥ 3. Statistical analysis was conducted using GraphPad Prism t test calculator and *p < 0.05, **p < 0.01, ***p < 0.001, ****p < 0.0001.

## Results and discussion

### Characterization of liposomes incorporating VP and gold nanoparticles

We prepared several types of liposome samples including liposomes incorporating VP and gold nanoparticles of two sizes 10 nm and 5 nm (Lipo-VP-10Au for 10 nm gold and Lipo-VP-5Au for 5 nm gold), liposomes incorporating VP (Lipo-VP) only and empty liposomes.

**Fig. 1:**
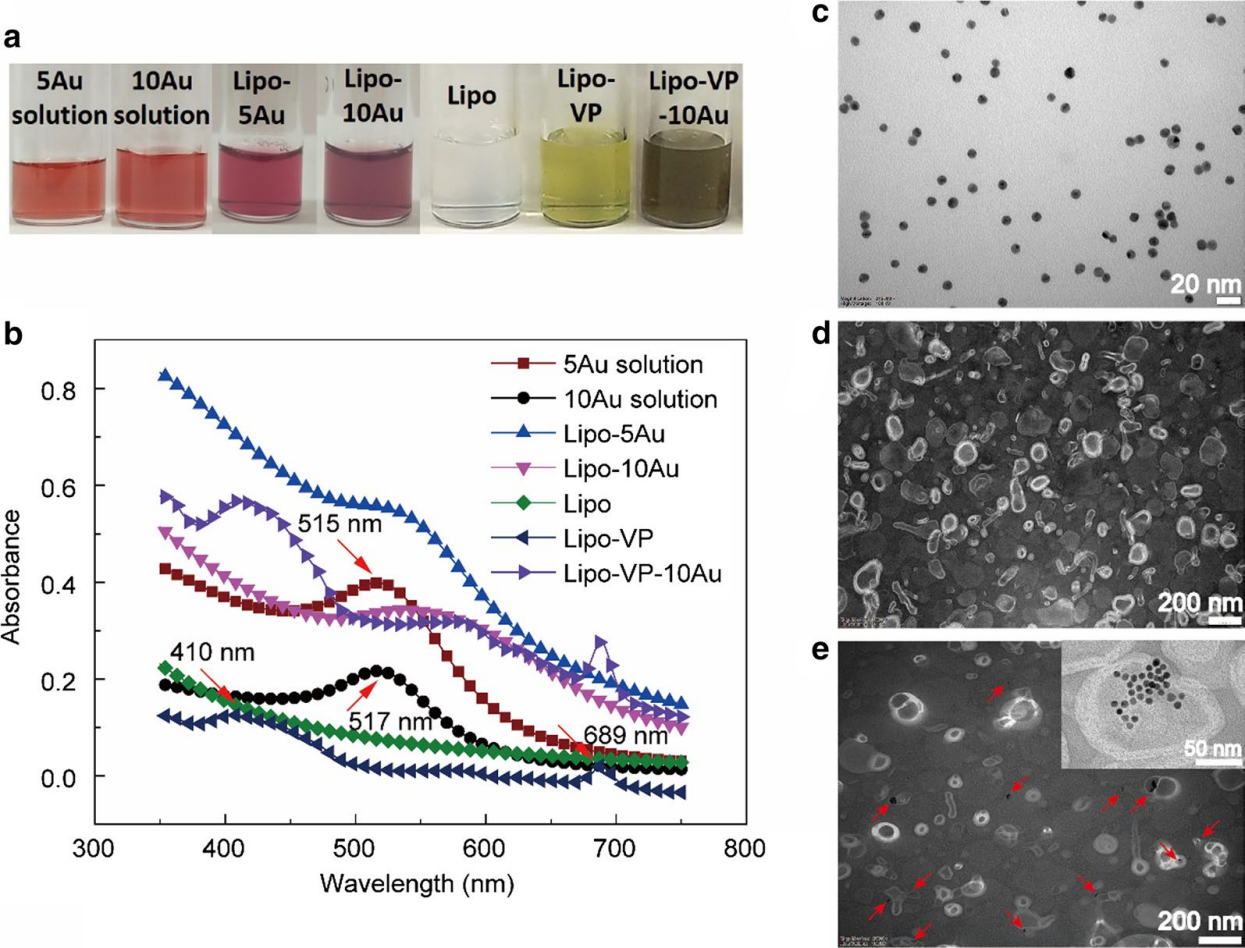
Characterisation of liposomes incorporating VP and gold nanoparticles. **a** Photograph of liposome samples and pure gold colloidal solution. **b** Absorption spectra of different liposome samples and pure gold colloidal solution. Red arrows indicated typical absorption peaks of VP (~ 410 nm and ~ 689 nm), 5 nm gold nanoparticles (~ 515 nm and 10 nm gold nanoparticles ~ 517 nm). **c**–**e** TEM images of 10 nm gold nanoparticles (**c**) pure liposomes (**d**) and liposomes loaded with 10 nm gold nanoparticles (**e**) Red arrows indicate gold nanoparticles encapsulated inside the liposomes

The sizes and zeta potential of as-prepared liposomes are summarised in Additional file 1: Fig. S1. Figure 1a and b shows photographs and the absorption spectra of pure gold colloidal solutions and different liposome samples. An obvious colour difference was observed between the pure gold colloidal solution and liposome-formulated gold (Fig. 1a). Such colour change from red to blue indicates the aggregation of gold nanoparticles when they were loaded inside liposomes, which is apparent in the red shift of the gold absorption peak shown in Fig. 1b. The absorption peak of 10 nm gold nanoparticles shifted from 517 nm in the colloidal solution to 537 nm in liposomes indicating some aggregation in the liposomal membranes. The absorption peaks of VP around 410 nm and 689 nm were observed in both Lipo-VP and Lipo-VP-10Au (Fig. 1b), confirming incorporation of VP, the peak location is consistent with published work [38]. We analysed the absorption spectra of pure TPP, Lipo-VP and TPP-Lipo-VP as shown Additional file 1: Fig. S1. The characteristic absorption peak of pure TPP is around 267 nm, which is also observed in the TPP-Lipo-VP sample.

The TEM image illustrate the morphology of liposomes confirming that the 10 nm gold nanoparticles were incorporated in the hydrophilic core (Fig. 1e). The TEM contrast is provided by higher electron density of gold compared with the liposomes. A similar TEM image of the liposomes incorporating 5 nm gold nanoparticles is shown in Additional file 1: Fig. S2 where gold nanoparticle clusters were observed.

### ^1^O_2_ generation under X-ray radiation

**Fig. 2:**
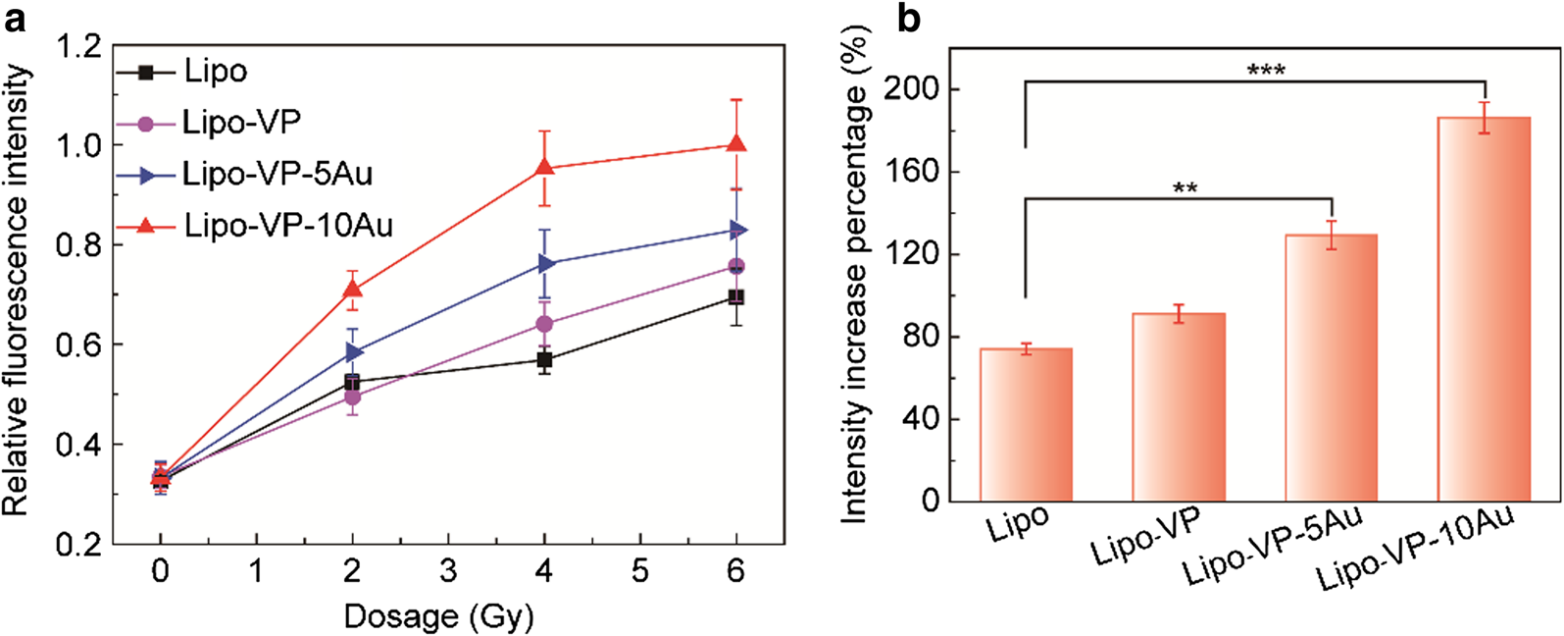
^1^O_2_ generation from different liposome samples under X-ray radiation. **a** Relative SOSG fluorescence intensities from liposome samples at different X-ray doses. **b** Percentage increase in SOSG fluorescence intensities from different liposome samples under 4 Gy X-ray radiation. Error bars show standard deviation from four measurements

Generation of cytotoxic ROS, such as ^1^O_2_ is a key factor responsible for the PDT effect. ^1^O_2_ generation in this work was determined by using the SOSG probe which produces a strong fluorescence signal at 525 nm for 488 nm excitation in the presence of ^1^O_2_ [24]. We confirmed ^1^O_2_ generation by monitoring the SOSG fluorescence intensity at 525 nm wavelength at different X-ray doses, as displayed in Fig. 2a. Among the tested liposomes loaded with 10 nm and 5 nm gold nanoparticles, those with 10 nm gold (Lipo-VP-10Au) produced the highest amount of ^1^O_2_, with a percentage increase of approximately 186% under X-ray radiation at 4 Gy (Fig. 2b). For 5 nm gold loaded samples (Lipo- VP-5Au), we observed a 129% increase in ^1^O_2_ enhancement, compared with liposomes with only VP (Lipo-VP) (91%). Such enhancement of ^1^O_2_ in the presence of gold nanoparticles is consistent with our previous work [24] and attributed to the following mechanism. As a heavy metal element, gold nanoparticles are well-known radiosensitizers, able to amplify the radiation doses in tumour tissue [39, 40]. As a consequence, the VP molecules in the presence of gold nanoparticles are able to interact more strongly with ionising radiation than the VP on its own, causing enhanced ^1^O_2_ generation (Fig. 2b).

### Intracellular ^1^O_2_ generation

**Fig. 3:**
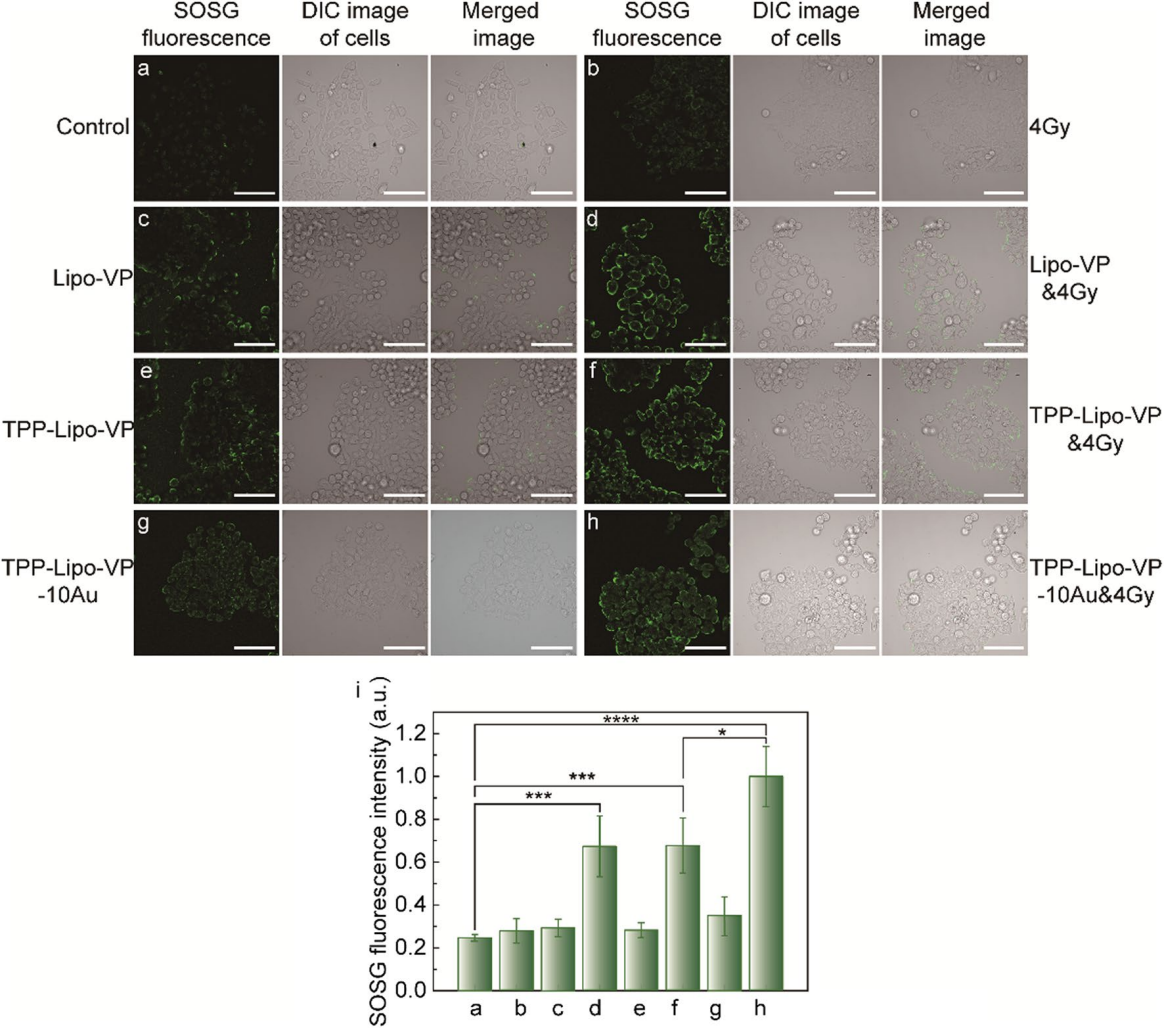
Intracellular ^1^O_2_ production from liposomes with and without X-ray radiation. (**a**–**h**) Representative confocal laser scanning microscopy images of SOSG in **a** HCT116 cells without any treatment, **b** HCT116 cells after 4 Gy X-ray irradiation, **c** HCT116 cells treated with Lipo-VP (250 µM) for 4 h, **d** HCT116 cells treated with Lipo-VP (250 µM) for 4 h and 4 Gy X-ray irradiation, **e** HCT116 cells treated with TPP-Lipo-VP (250 µM) for 4 h, **f** HCT116 cells treated with TPP-Lipo-VP (250 µM) for 4 h and 4 Gy X-ray irradiation, **g** HCT116 cells treated with Lipo-VP-10Au (250 µM) for 4 h and **h** HCT116 cells treated with Lipo-VP-10Au (250 µM) for 4 h and 4 Gy X-ray irradiation. Scale bar is 70 µm. **g** Quantitative analysis of ^1^O_2_ generation in HCT116 cells at different treatment conditions

After confirmation of ^1^O_2_ generation in solution, we assessed the intracellular ^1^O_2_ generation under different treatment conditions (liposomes ± X-ray radiation). As shown in Fig. 3a and b, the SOSG fluorescence intensity observed in the cells treated with 4 Gy X-ray radiation did not show significant differences compared with the untreated control cells (p = 0.32). This suggests that the dose of 4 Gy is safe for the cells use in this study. A similar amount of ^1^O_2_ was found in the cell groups respectively treated with Lipo-VP, TPP-Lipo-VP and TPP-Lipo-VP-10Au alone (Fig. 3c, e and g). Also, no significant differences were observed compared with the control group (p = 0.07, 0.11 and 0.07, respectively). This indicates that minimal ^1^O_2_ was generated by the empty liposomes in these conditions.

However, a clearly higher ^1^O_2_ production was observed in the cells treated with X-ray triggered liposome samples (Fig. 3d, f and h). Among these samples, TPP-Lipo–VP-10Au in combination with 4 Gy generated the highest amount of ^1^O_2_.

In addition, we checked the intracellular ^1^O_2_ production under varying X-ray radiation dose. As shown in Additional file 1: Fig. S3, even under 6 Gy radiation alone, the ^1^O_2_ generation was still lower than in the cells treated with Lipo-VP-10Au in combination with 4 Gy (Fig. 3h). All these findings indicate that intracellular ^1^O_2_ generation is induced by the interaction between X-rays and a combination of VP and gold nanoparticles have augmented this effect.

### Mitochondria localization of TPP-conjugated liposomes

Mitochondria have been proposed to be the most effective subcellular targets in PDT since ROS-induced mitochondrial DNA damage results in augmented cytotoxicity in PDT [9]. Therefore, it would be desirable for photosensitisers or their delivery vehicles to be accumulated within or in close proximity to mitochondria. To achieve this, we conjugated the liposomes with a mitochondria-targeting moiety, TPP using the EDC-NHS coupling method. The mitochondria-targeting capabilities of the TPP-Lipo-VP were investigated by incubating the targeted liposomes loaded with VP alone (TPP-Lipo-VP) with HCT116 cells and using non-targeted Lipo-VP as controls.

**Fig. 4:**
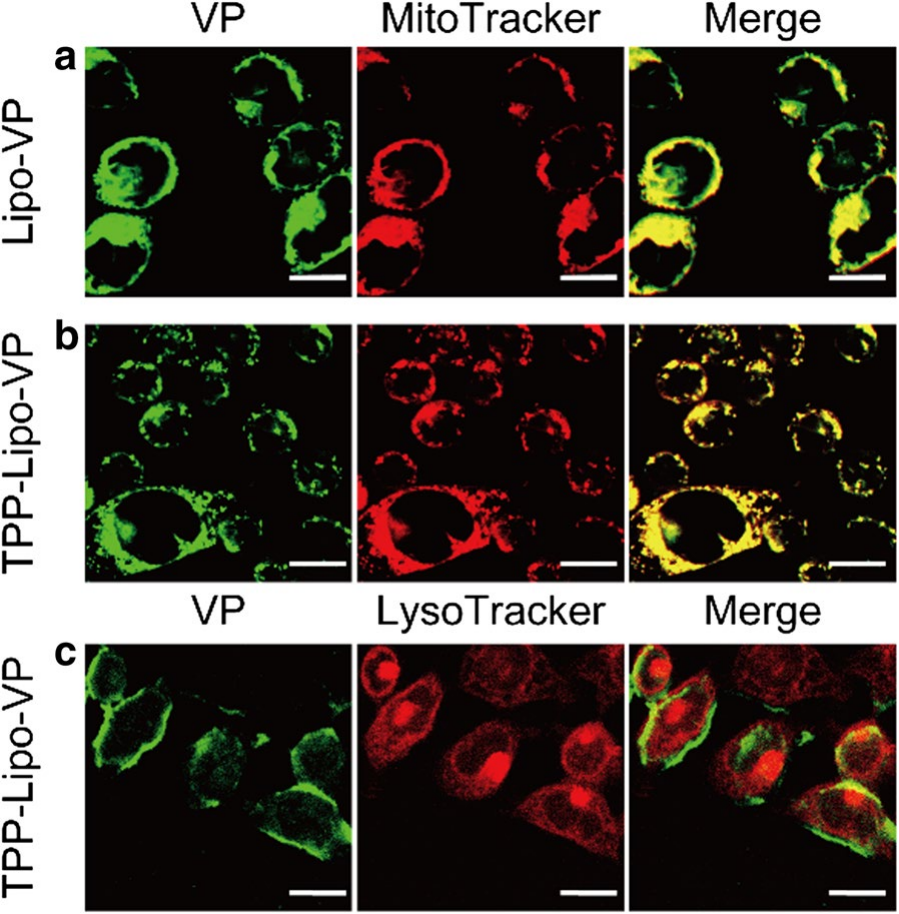
Colocalization of Lipo-VP and TPP-Lipo-VP with the mitochondria and lysosomes. **a**–**c** Representative confocal laser scanning microscopy images of VP (pseudo green colour to distinguish between VP and trackers), Mitotracker Red (red channel) and LysoTracker (red channel) in HCT116 cells after incubation with Lipo-VP (250 µM) and TPP-Lipo-VP (250 µM). Merged images of green and red channels indicated colocalization level of the liposomes and mitochondria (or lysosomes). Scale bar is 20 µm

As shown in Fig. 4a and b, cells treated with TPP-Lipo-VP showed a higher liposomal uptake than non-conjugated liposomes Lipo-VP in the mitochondria. In this figure we are relying on intrinsic fluorescence of VP. Quantitative analysis using the ImageJ/Fiji “colocalization analysis” function also revealed a higher degree of overlap of the TPP-conjugated liposomes with MitoTracker in the mitochondria of the cells (Pearson’s correlation coefficient, PCC = 0.63), compared with the non-conjugated liposomes (PCC = 0.53). This indicated that the TPP-conjugated liposomes have a greater mitochondrial selectivity than non-targeted ones. In addition, TPP-Lipo-VP showed almost no colocalization with the lysosomes labelled by Lysotracker (PCC = − 0.03, Fig. 4c), indicating these TPP-modified liposomes were, in fact, preferentially accumulated in mitochondria due to the capability of TPP for mitochondrial targeting. These findings are consistent with other reports on the localization of TPP-modified nanoparticles to the mitochondria of pancreatic cancer, endothelial, and prostate cancer cells [41, 42].

Based on these data we selected TPP-conjugated liposomes as the therapeutic agent for further studies. We investigated the cellular uptake of TPP-Lipo-VP in the HCT116 cells by incubating the cells with TPP-Lipo-VP for 1 h, 2 h and 4 h, respectively. As shown in Additional file 1: Fig. S4a, the pseudo green colour from VP was clearly observed after 2 h and 4 h incubation time, compared with 1 h confirming cellular uptake of TPP-Lipo-VP which appears to reach saturation after 2 h treatment. Consistently, we found no significant difference in VP fluorescence intensity between 2 h and 4 h incubation via flow cytometry measurements (Additional file 1: Fig. S4b), therefore we chose 2 h incubation time for further cellular experiments.

### Alterations in the mitochondrial membrane potential

**Fig. 5:**
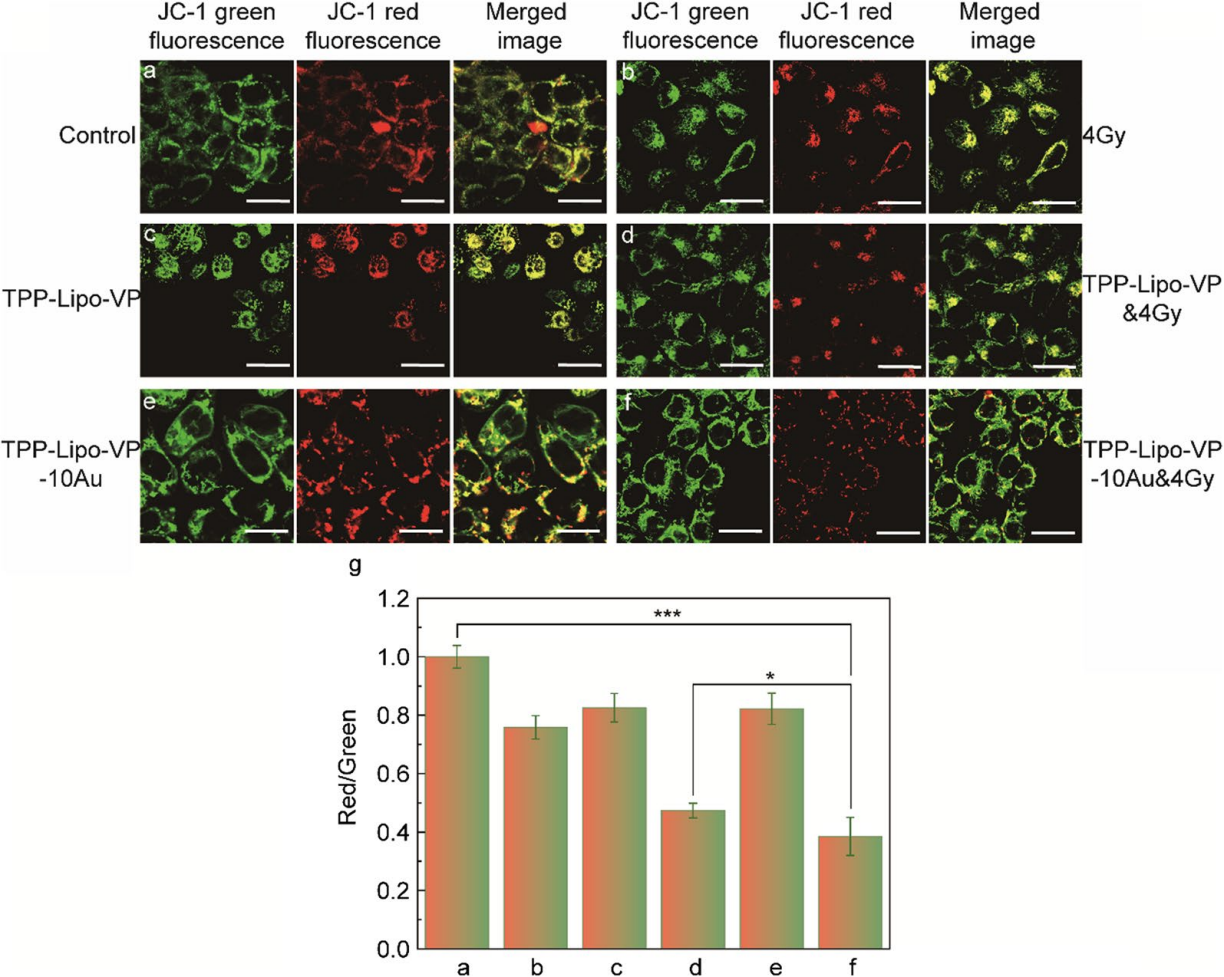
Changes in JC-1 fluorescence signal (Δ*ψ*_m_) in HCT116 cells under different treatment conditions: **a** the cells without any treatment, **b** the cells treated with 4 Gy X-ray radiation, **c** the cells treated with TPP-Lipo-VP (250 µM), **d** the cells treated with TPP-Lipo-VP (250 µM) and 4 Gy X-ray radiation, **e** the cells treated with TPP-Lipo-VP-10Au (250 µM) and **f** the cells treated with TPP-Lipo-VP-10Au (250 µM) and 4 Gy X-ray radiation. Scale bar is 20 µm. The high-membrane potential resulted in the JC-1 aggregation in the membrane, as shown by the strong red fluorescent signal, and low membrane potential caused the monodispersed release of JC-1 to the cytosol, as shown by the strong green fluorescent signal. **g** Red-to-green channel ratio determines the rate of membrane potential decay and cells dead

To verify whether the mitochondrial function was affected by X-ray induced PDT using our liposomes, the mitochondrial membrane potential was examined by JC-1 staining and confocal microscopy. The JC-1 probe was used to evaluate the changes in Δ*ψ*_m_ upon liposome administration. The JC-1 dye is aggregated in normal mitochondrial membrane causing the dye to emit red fluorescence. By contrast, in the unhealthy or damaged mitochondria JC-1 monomers localise in the membrane at low concentrations, producing green fluorescence. Thus, the ratio of JC-1 aggregates (red signal) to monomers fluorescence (green signal) can be used as an indicator to analyse Δ*ψ*_m_. As shown in Fig. 5a, c and e, the JC-1 signal was not obviously changed in the cells treated with liposomes (TPP-Lipo-VP or TPP-Lipo-VP-10Au), compared with the control group. This indicates minimal toxicity of the liposomes in HCT116 cells under the experimental conditions. However, when we combined the aforementioned liposomes with X-ray radiation, the cells presented a weaker red signal due to mitochondrial depolarization induced by the cytotoxic singlet oxygen generated by the X-ray induced PDT effect (Fig. 5d and f). In particular, the cells treated with TPP-conjugated liposomes exposed to X-rays exhibited the largest Δ*ψ*_m_ damage (the smallest red/green ratio in Fig. 5g), which was consistent with our previous data for the intracellular ^1^O_2_ production (Fig. 3f).

### In vitro cytotoxicity

**Fig. 6:**
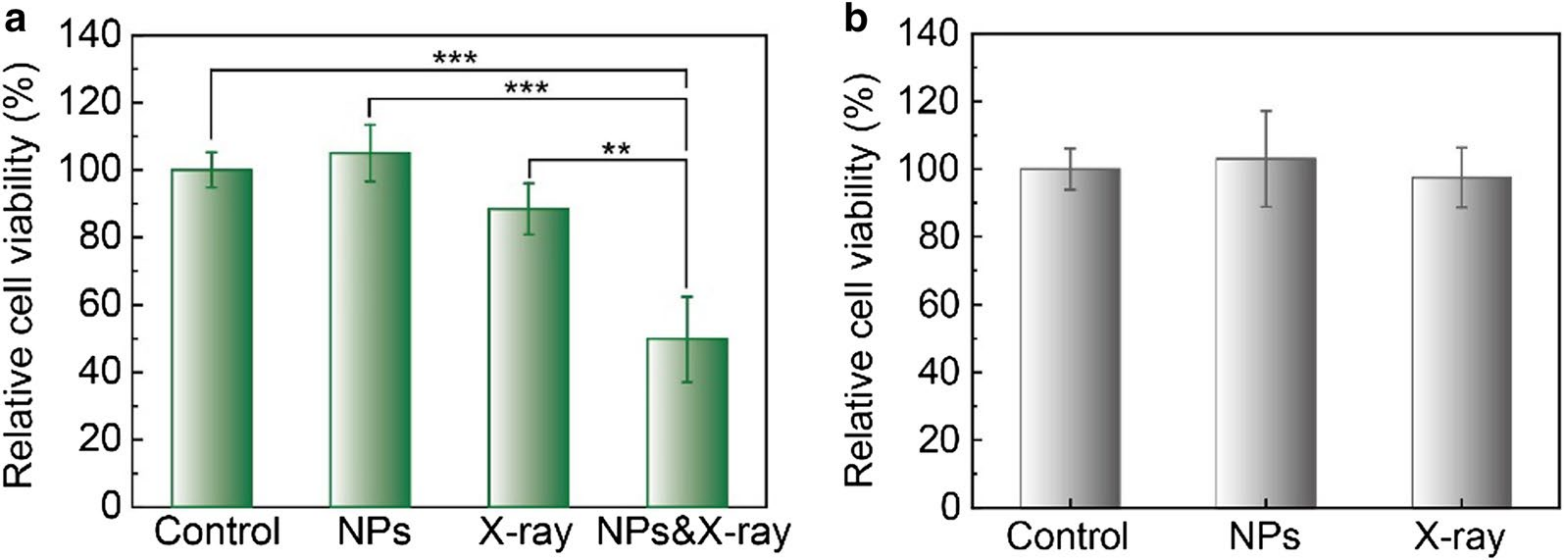
**a** In vitro cytotoxicity of TPP-Lipo-VP-10Au in HCT116 cells after X-ray irradiation. The cells were treated with X-ray alone, TPP-Lipo-VP-10Au (250 µM) alone and combined condition. **b** The dark cytotoxicity of TPP-Lipo-VP-10Au (250 µM) and X-ray irradiation (4 Gy) in CCD841 CoN cells

We further assessed the cytotoxicity of the in vitro X-ray induced PDT effect in HCT116 cells. Cell viability assays under various treatments were performed to estimate the potential cytotoxicity effect on HCT116 by using the MTS assay. Both TPP-Lipo-VP-10Au (p = 0.4972) and the X-ray radiation alone (p = 0.1334) induced low cytotoxicity in HCT116 cells and normal colon CCD 841 cells (p = 0.7342), with minimal changes to cell viability compared to the control group (Fig. 6). However, when these two treatments were combined, a significant decrease in HCT116 cell survival was observed (49.8%). This enhanced cytotoxicity effect is attributed to efficient production of ^1^O_2_ from the activated VP under X-ray radiation augmented by ROS generation by gold nanoparticles under these conditions.

### In vitro X-ray induced PDT effect and apoptosis/necrosis assays

**Fig. 7:**
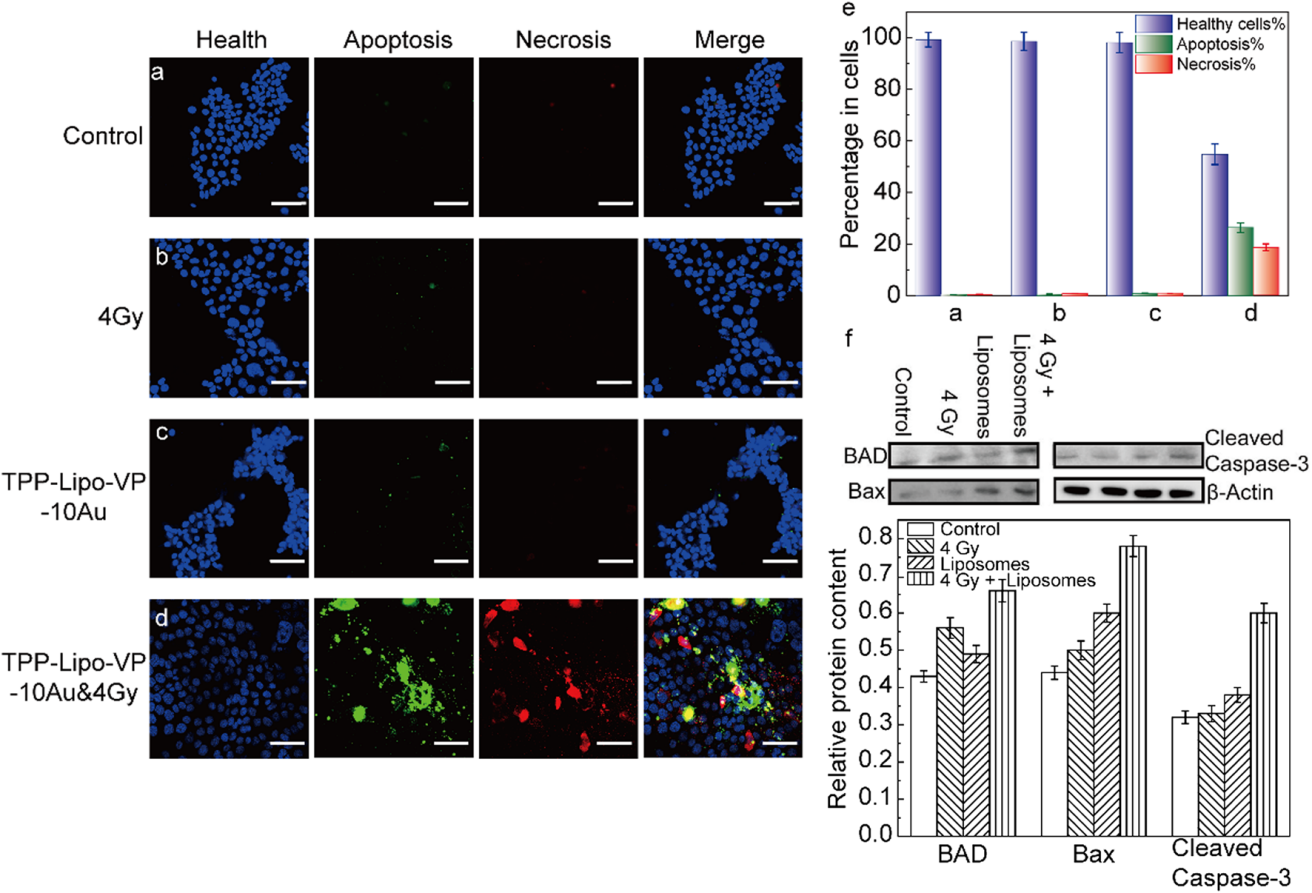
Apoptosis/necrosis assays performed in HCT116 cells at 24 h after the treatments. **a–d** Representative confocal laser scanning microscopy images of the cells after different treatments as indicated. The concentration of TPP-Lipo-VP-10Au used to treat the cells was 250 µM. Scale bar is 70 µm. **e** Relative changes in levels of apoptosis and necrosis upon different treatments as indicated in **a**–**d**. **f** Relative protein expression was analysed by using ImageJ software. Protein expression was normalized by use of the loading control protein (β-actin)

The apoptosis/necrosis assays were also carried out to investigate the cell death pathways under our treatment conditions. Under X-ray radiation alone and TPP-Lipo-VP-10Au alone, the cells showed a low-level apoptosis and necrosis, compared with the controls (Fig. 7a, b and c). This indicated that the lethality of low dose X-ray and the liposomes with the current concentration is negligible. By contrast, after the treatment with TPP-Lipo-VP-10Au and 4 Gy, the percentage of apoptotic and necrotic cells significantly increased to about 26% and 19%, respectively. Correspondingly, the percentage of healthy cells reduced from 99% in the control to 55% in the cells treated with (Fig. 7d). Taken together, these findings indicate that mitochondria-targeted liposomes displayed the higher in vitro X-ray induced PDT effect. To further elucidate the mechanism of PDT-mediated apoptosis, we investigated the expression of apoptosis-related proteins in HCT116 cells using western blotting. Bax and BAD regulate apoptosis mainly at the level of the mitochondria [43]. Caspase is also an important apoptosis inducer that exists on the outer membranes of mitochondria [44]. As shown in Fig. 7f, increased expression of Bax and BAD as well as activated caspase-3 was observed in the cells treated with TPP-conjugated liposomes in combination with 4 Gy radiation, indicating that X-ray induced PDT led to cell apoptosis mainly via the mitochondrial pathways.

## Conclusions

We have developed a novel mitochondrially-targeted liposome delivery system for an enhanced X-ray PDT effect in deep tissue. The liposomes were formulated by co-embedding VP and gold nanoparticles in the same liposomal platform and then they were conjugated with TPP to target mitochondria. These liposomes exhibited a mitochondria-targeting capacity and they enhanced ^1^O_2_ generation in HCT116 cells under X-ray radiation at 4 Gy. We have also observed X-ray PDT-induced mitochondrial damage and elucidated the mechanisms of cancer cell death. Together, these results show the mitochondria-targeted and X-ray triggerable liposome delivery system could provide new options beyond traditional PDT for deep tumour therapy.

## Supplementary information

**Additional file 1: Figure S1.** Absorption spectra of different liposome samples and pure TPP solution. **Figure S2. (a)** Size and Zeta potential distribution determined by dynamic light scattering. TEM images of **(b)** 5 nm gold nanoparticles and **(c)** the liposomes loaded with 5 nm gold nanoparticles. **Figure S3.** Intracellular ^1^O_2_ production under X-ray radiation at different doses. **(a)** Representative confocal fluorescence images of SOSG in HCT116 cells after the treatment with X-ray triggered liposomes. Scale bar is 70 µm. **(b)** Quantitative analysis of ^1^O_2_ generation in HCT116 cells (*n *= 4). **Figure S4.** Cellular uptake of TPP-Lipo-VP in HCT 116 cells. **(a)** Representative confocal laser scanning microscopy images of HCT 116 cells incubated with TPP-Lipo-VP (250 µM) for 1 h, 2 h and 4 h, respectively. Scale bar is 20 µm. **(b)** Quantitative analysis of VP fluorescence intensity via flow cytometry

## Data Availability

Not applicable.
