# Supplementary material for: X-ray induced photodynamic therapy (PDT) with a mitochondria-targeted liposome delivery system
Source: J Nanobiotechnology. 2020 Jun 10;18:87. doi: 10.1186/s12951-020-00644-z (PMC7288491; doi:10.1186/s12951-020-00644-z)
Supplement: Supplementary file 1 — Additional file 1: Figure S1. Absorption spectra of different liposome samples and pure TPP solution. Figure S2. (a) Size and Zeta potential distribution determined by dynamic light scattering. TEM images of (b) 5 nm gold nanoparticles and (c) the liposomes loaded with 5 nm gold nanoparticles. Figure S3. Intracellular 1O2 production under X-ray radiation at different doses. (a) Representative confocal fluorescence images of SOSG in HCT116 cells after the treatment with X-ray triggered liposomes. Scale bar is 70 µm. (b) Quantitative analysis of 1O2 generation in HCT116 cells (n = 4). Figure S4. Cellular uptake of TPP-Lipo-VP in HCT 116 cells. (a) Representative confocal laser scanning microscopy images of HCT 116 cells incubated with TPP-Lipo-VP (250 µM) for 1 h, 2 h and 4 h, respectively. Scale bar is 20 µm. (b) Quantitative analysis of VP fluorescence intensity via flow cytometry [file 12951_2020_644_MOESM1_ESM.docx]

**Additional file**

**X-ray triggered** **photodynamic therapy (PDT) via** **mitochondria-targeted**

**liposome delivery system**

Xuefan Gu, ^a,b,c^ Chao Shen, ^c^ Hua Li, ^a^ Ewa M Goldys, ^*b^ and Wei Deng ^*b^

^1^College of Chemistry and Chemical Engineering, Xi'an Shiyou University, Xi'an, 710065, China.

^2^the Graduate School of Biomedical Engineering, University of New South Wales, Sydney, Kensington, 2052 NSW, Australia.

^3^Faculty of Science and Engineering, Macquarie University, Sydney, 2109 NSW, Australia.

**
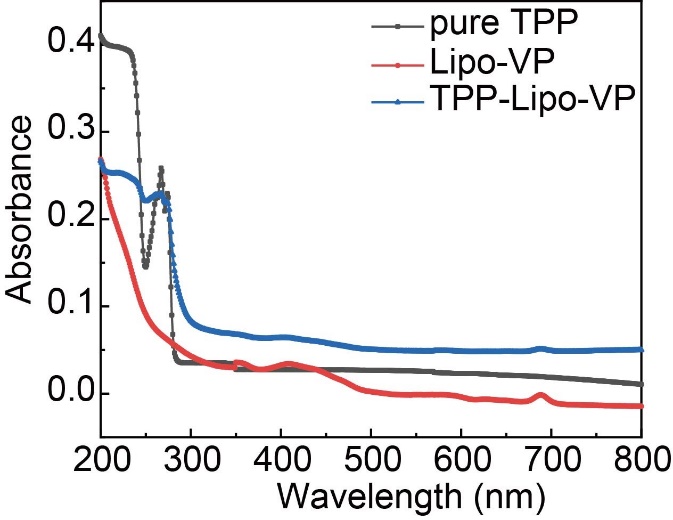
**

**Figure S1** Absorption spectra of different liposome samples and pure TPP solution.

**
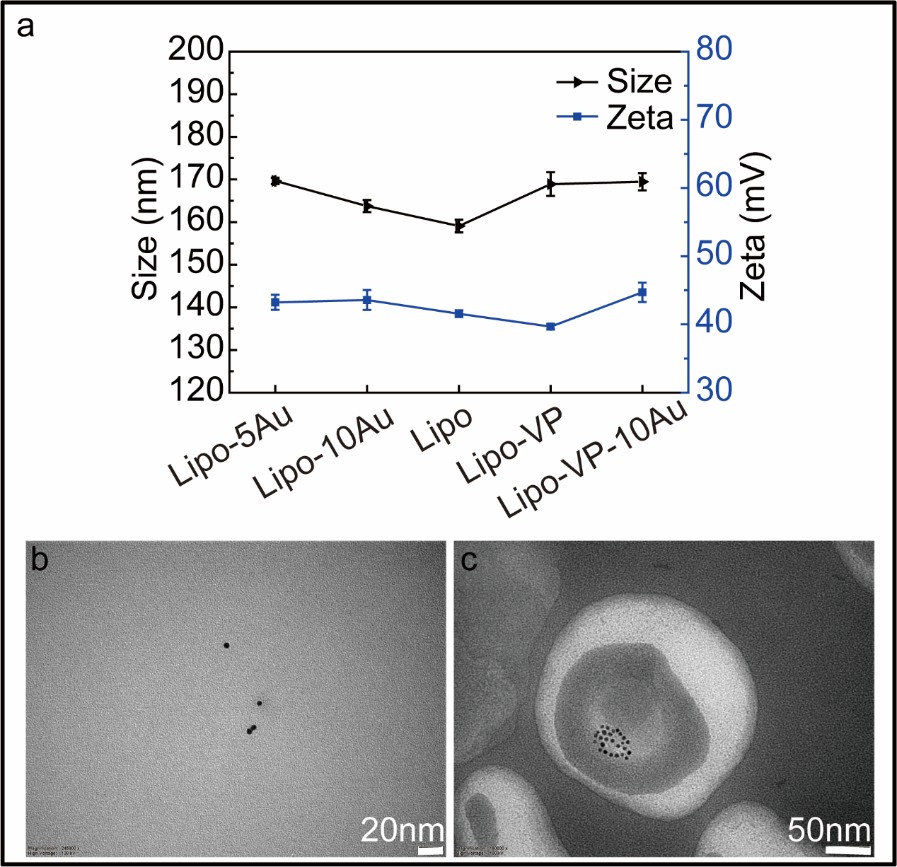
**

**Figure S2 (a)** Size and Zeta potential distribution determined by dynamic light scattering. TEM images of **(b)** 5 nm gold nanoparticles and **(c)** the liposomes loaded with 5 nm gold nanoparticles.
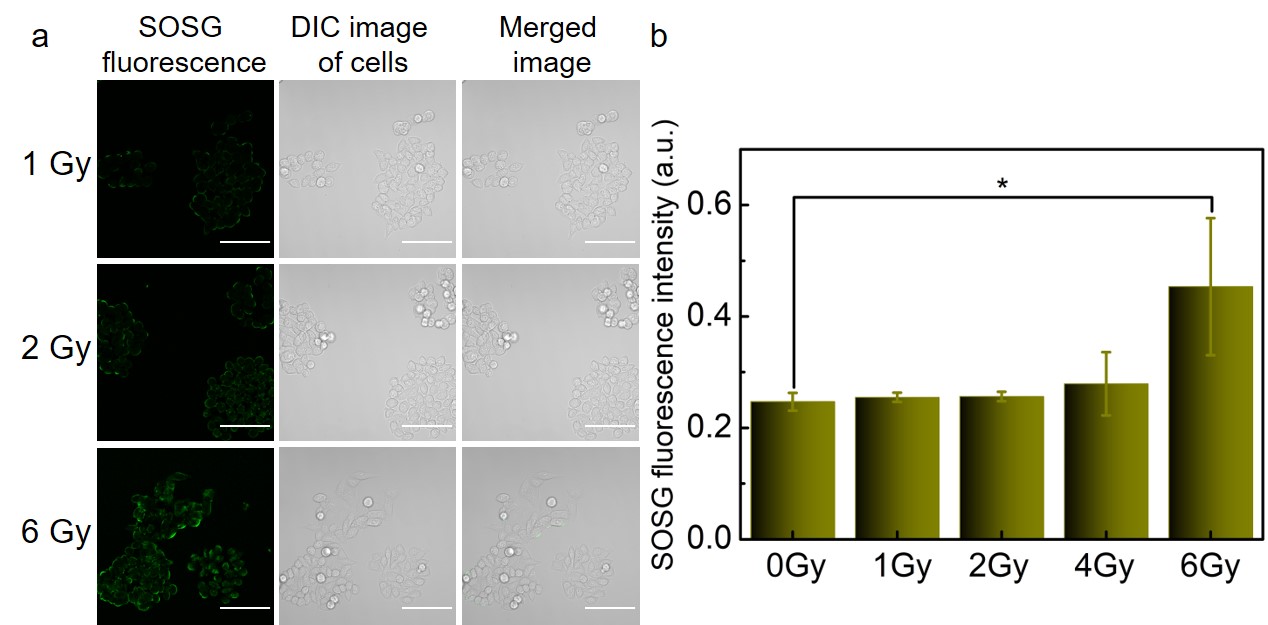


**Figure S3** Intracellular ^1^O_2_ production under X-ray radiation at different doses. **(a)** Representative confocal fluorescence images of SOSG in HCT116 cells after the treatment with X-ray triggered liposomes. Scale bar is 70 µm. **(b)** Quantitative analysis of ^1^O_2_ generation in HCT116 cells (*n*=4)


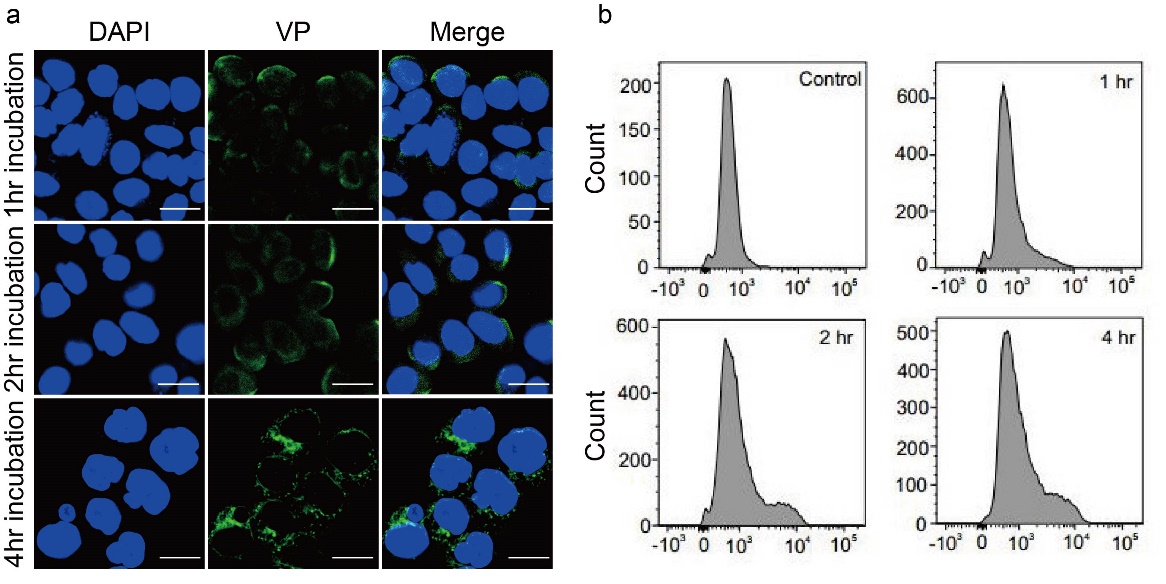


**Figure S4** Cellular uptake of TPP-Lipo-VP in HCT 116 cells. **(a)** Representative confocal laser scanning microscopy images of HCT 116 cells incubated with TPP-Lipo-VP (250 µM) for 1 h, 2 h and 4 h, respectively. Scale bar is 20µm. **(b)** Quantitative analysis of VP fluorescence intensity via flow cytometry.
